# Clinical relevance of the 21-gene Recurrence Score^®^ assay in treatment decisions for patients with node-positive breast cancer in the genomic era

**DOI:** 10.1038/s41523-018-0082-6

**Published:** 2018-08-20

**Authors:** Eleftherios P. Mamounas, Christy A. Russell, Anna Lau, Michelle P. Turner, Kathy S. Albain

**Affiliations:** 1 Orlando Health UF Health Cancer Center, 1400 S. Orange Ave., Orlando, FL 32806 USA; 2 Genomic Health, Inc., 301 Penobscot Dr., Redwood City, CA 94063 USA; 3 Loyola University Chicago Cardinal Bernardin Cancer Center, 2160 S. First Ave., Maywood, IL 60153 USA

## Abstract

In contemporary management of early-stage breast cancer, clinical decisions regarding adjuvant systemic therapy are increasingly made after considering both genomic assay results and clinico-pathologic features. Genomic information augments the prognostic information gleaned from clinico-pathologic features by providing risk estimates for distant recurrence and/or breast cancer-specific survival based on individual tumor biology. The 21-gene Oncotype DX Breast Recurrence Score^®^ (RS) assay is validated to be prognostic and predictive of chemotherapy benefit in patients with hormone receptor-positive (HR+), HER2-negative early-stage breast cancer, regardless of nodal status. Because patients frequently are recommended to receive adjuvant chemotherapy based on the perceived poor prognosis related to a positive nodal status, inconsistent use of any prognostic genomic assay in the node-positive (N+) setting likely results in overtreatment of some patients, particularly those with a low genomic risk as defined by the RS test. This comprehensive review of the evidence for the RS assay in patients with N+, HR+, HER2-negative early-stage breast cancer focuses on outcomes of patients with low RS results treated with hormonal therapy alone. Aggregate findings show that the RS assay consistently identifies patients with low genomic risk N+ breast cancer, in whom adjuvant chemotherapy can be avoided without adversely affecting outcomes. This evidence suggests that HR+ patients with limited nodal involvement and low RS results should discuss with their physicians the pros and cons of adjuvant chemotherapy at the time their treatment plans are being decided.

## Introduction

Whether to use adjuvant chemotherapy is an important decision that clinicians and patients face following diagnosis of invasive breast cancer and the completion of primary surgical treatment. In the absence of tumor-specific genomic information, systemic adjuvant treatment decisions largely rely on prognostic information gleaned from clinical and pathologic features of the patient and tumor. Younger patient age, larger tumor size, higher tumor grade, and positive nodal status are each associated with poorer prognosis, including the higher risk of distant recurrence, with lymph node status being the strongest contributor.^1–3^ In addition, positive hormone receptor status and HER2 status are both prognostic and predictive of treatment response.^4,5^ Patients with high-risk clinico-pathologic features—such as positive nodal status—are generally recommended to receive adjuvant chemotherapy.

Previous clinical trials in hormone receptor-positive (HR+), HER2-negative, node-positive (N+) breast cancer showed that chemotherapy plus hormonal therapy yielded better outcomes than hormonal therapy alone.^6–8^ In a meta-analysis, the Early Breast Cancer Trialists’ Collaborative Group (EBCTCG) showed that this benefit of chemotherapy is largely independent of age, nodal status, tumor differentiation, or estrogen receptor (ER) level. The meta-analysis, however, was unable to demonstrate the proportional benefit of chemotherapy in those patients with ER+, HER2-negative invasive breast cancer.^7^ With these findings, one cannot assign an even proportional risk reduction for chemotherapy across all prognostic groups. These observations indicate that the prognostic power of nodal status has a limit in the capacity to predict chemotherapy benefit, and that population-level prognostication and treatment benefit will not relate to the prognosis and treatment benefit expected for an individual patient. Therefore, universal treatment of patients with HR+, HER2-negative, N+breast cancer with adjuvant chemotherapy may represent overtreatment for many.

The advent of genomic assays for use in ER+ early breast cancer has accelerated a functional understanding of the disease. Results of assays providing prognostic genomic information have been accepted as complementary to other prognostic information, as evidenced by the incorporation of genomic assays into clinical practice guidelines put forth by international agencies.^9–14^ Importantly, the risk of distant recurrence and/or breast cancer-specific mortality (BCSM) estimated by genomic assays is based on tumor biology, thereby individualizing prognostication to the level of the patient.

The 21-gene Oncotype DX Breast Recurrence Score^®^ (RS) test (Genomic Health, Inc.; Redwood City, CA) is an RT-PCR-based test that measures the activity of 21 genes (16 cancer-related; 5 reference).^15^ The test generates a Recurrence Score^®^ result that represents an individualized estimate of risk of distant recurrence and/or BCSM (prognosis)^16,17^ and predicts the likelihood of adjuvant chemotherapy benefit.^18–20^ The cumulative findings of the National Surgical Adjuvant Breast and Bowel Project (NSABP) B-20, the Trial Assigning IndividuaLized Options for Treatment (TAILORx), and Southwest Oncology Group (SWOG) S8814 trials have shown that there is a clear interaction between chemotherapy benefit and the 21-gene test.^18–20^ These findings have demonstrated a lack of benefit of chemotherapy when added to endocrine therapy for patients with low RS results and a substantial benefit for those women with high RS results.^18–20^ Although the 21-gene test is considered the standard-of-care in the HR+, HER2-negative, node-negative setting to guide adjuvant chemotherapy decisions,^9–14^ it is not as uniformly utilized in the N+ setting.^11,21^ There exists a discordance in practice guidelines in N+ breast cancer that stems from a differing emphasis on levels of evidence and timing of the guideline group publications. This has resulted in a lack of uniform acceptance of the available genomic assays in the US by insurance companies and internationally by health policies in different countries. The lack of access to genomic assays, and specifically the 21-gene test will likely result in the overtreatment of many patients, namely, those with tumors that are biologically unlikely to respond to chemotherapy. Here, we present a summary of the large body of evidence supporting the clinical value of the 21-gene test to guide systemic adjuvant treatment decisions in patients with HR+, HER2-negative, N+ breast cancer. We focus on studies that included at least one cohort that received hormonal therapy only in order to assess outcomes of patients with low RS results who received no adjuvant chemotherapy.

## Summary of evidence for the RS test in HR+, HER2-negative, N+ breast cancer

Five studies are considered in this review. Two are validation studies that used a prospective-retrospective design,^16,18,22^ one is an outcomes-based, prospectively designed clinical trial,^23–26^ and two are population-based registries that followed patient outcomes in a prospective manner.^27–29^ Descriptions of the studies included in the review are shown in Table 1.

**Table 1:** Studies included in this analysis of the Recurrence Score assay in node-positive breast cancer (*N* = 9055)

Study^a^	Years of study enrollment^b^	Type of study	*N*	Study design	Primary endpoint(s)	Years of follow-up
SWOG S8814^[18]^	1989–1995	Prospective-retrospective; validation	367 (62%/38% 1–3N+/≥4N+)	TAM vs. CAF→T	DFS BCSS	10 years
transATAC^[16]^	1996–2000	Prospective-retrospective; validation	306 (79%/21% 1–3N+/≥4N+)	ANA vs. TAM vs. ANA + TAM	DR	9 years
SEER^[27,28]^	2004–2013	Prospective outcomes	6768 (42%/54%/4% N1mi/1–3N+/≥4N+)	Population-based registry	BCSS	5 years
Clalit^[29]^	2006–2011	Prospective outcomes	709 (42%/58% N1mi/1–3N+)	Population-based registry	DR; BCSM	5 years
WSG PlanB^[23–26]^	2009–2011	Prospective outcomes	905 (100% 1–3N+)	RS <12: ET RS ≥12: CT	DFS; DDFS	5 years

*ANA* anastrozole, *BCSM* breast cancer-specific mortality, *BCSS* breast cancer-specific survival, *CAF* cyclophosphamide, doxorubicin, 5-fluorouracil, *CT* chemotherapy, *DDFS* distant disease-free survival, *DFS* disease-free survival, *DR* distant recurrence, *ET* endocrine therapy, *T or TAM* tamoxifen

^a^Numbers in brackets denote reference citations

^b^For prospective-retrospective studies, this refers to the time period when the parent study enrolled patients

**Fig. 1:**
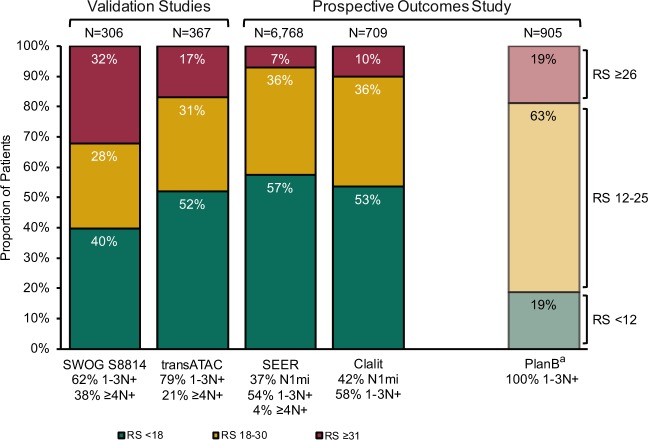
Recurrence Score distribution among studies in this analysis of the Recurrence Score assay in node-positive breast cancer (*N* = 9055). ^a^The WSG PlanB study enrolled patients with pN0 and pN1-3 breast cancer, but this analysis includes only those with 1–3N+; RS cut-points used were non-standard: RS <12, RS 12–25, and RS ≥26

The distribution of patients across RS groups in each study is shown in Fig. 1. Among the four studies that used standard RS cut-offs (RS <18, 18–30, ≥31), the single largest RS group was the RS <18 group. The PlanB study used non-standard RS cut-offs (RS <12, 12–25, >25), and the largest group in this study was the RS 12–25 group. The RS result distribution shows that a considerable proportion of patients have low-risk genomic signature breast cancer despite positive nodal status.

### Validation of prognostic utility of the RS test in HR+, HER2-negative, N+ breast cancer

The transATAC was a prospectively planned translational study of archival samples from the Arimidex, Tamoxifen, Alone or in Combination (ATAC) trial, in which postmenopausal patients with invasive breast cancer were randomized to anastrozole, tamoxifen, or the combination.^16,30^ The purpose of the transATAC study was to determine whether the RS result provided independent information on the risk of distant recurrence in the two monotherapy arms of ATAC. Of 9366 patients enrolled in the parent ATAC trial, 1231 tumor samples from the two monotherapy arms were evaluable in the transATAC study, including 306 samples from patients with HR+, HER2-negative, N+ breast cancer (*N* = 243 with 1–3N+; *N* = 63 with ≥4N+). The risk of distant recurrence among patients with N+ breast cancer (*N* = 306) differed significantly by RS group: mean 9-year risk of distant recurrence was 17% with RS <18, 28% with RS 18–30, and 49% with RS ≥31 (*p* < 0.001; Table 2) (unfortunately, the 9-year risk of distant recurrence was not provided in numerical form for those with 1–3N+ vs. ≥4N+). For any RS result, the risk of distant recurrence was found to be higher for patients with ≥4N+ than with 1–3N+. Notably, patients with 1–3N+ breast cancer and low RS results had estimates of 9-year risk of distant recurrence that were comparable to those of patients with node-negative breast cancer.^16^

**Table 2:** Summary of results from validation studies (level 1B) and the WSG PlanB study (level 1A)

	Standard RS cut-offs		
	RS <18	RS 18–30	RS ≥31
SWOG S8814 (N+; *N* = 367)			
*N* (%)	146 (40%)	103 (28%)	118 (32%)
% CT/% no CT	62%/38%	55%/45%	60%/40%
10-y DFS; HR (95% CI) for CT + HT vs. HT	1.02 (0.54, 1.93)	0.72 (0.39, 1.31)	0.59 (0.35, 1.01)
*p*-value	0.97	0.48	0.03
transATAC (N+; *N* = 306)			
*N* (%)	160 (52%)	94 (31%)	52 (17%)
% CT/% no CT	0%/100%	0%/100%	0%/100%
9-y DR (95% CI)	17% (12%, 24%)	28% (20%, 39%)	49% (35%, 64%)
*p*-value	<0.001 (RS results associated with risk of DR)		
	**Non-standard RS cut-offs**		
	**RS <12**	**RS 12–25**	**RS >25**
PlanB (1–3N+; *N* = 905)			
*N* (%)	170 (19%)^a^	567 (63%)	168 (19%)
% CT/% no CT	0%/100%^a^	100%/0%	100%/0%
5-y DFS (95% CI)	94.4%^a^ (89.5%, 99.3%)	94.3% (91.9%, 96.7%)	83.6% (77.1%, 90.1%)
*p*-value	<0.001		
5-y DDFS	97.9%	96.0%	86.0%
*p*-value	<0.001 for RS >25 vs. RS <12 or RS 12–25		

*CI* confidence interval, *CT* chemotherapy, *DFS* disease-free survival, *DR* distant recurrence, *HR* hazard ratio, *HT* hormonal therapy, *RS* Recurrence Score result

^a^Of 170 patients with 1–3N+ breast cancer and RS <12, 110 patients received hormonal therapy alone; the remaining 60 patients received chemotherapy. Data shown here are for the 110 patients with 1–3N+ breast cancer and RS <12 who received hormonal therapy alone

The prospective West German Study Group (WSG) PlanB trial was designed to assess the difference in disease-free survival (DFS) for women with HER2-negative, high-risk lymph node-negative, or lymph node-positive breast cancer using an anthracycline or a non-anthracycline adjuvant chemotherapy regimen. A total of 3198 women were enrolled in the trial. An early amendment resulted in the testing of all patients with ER+ breast cancer who had node-negative or 1–3N+ disease.^26,31^ Those with ER+, pN0 or pN1 disease, and RS <12 received endocrine therapy only (after a protocol amendment) and those with RS ≥12 were randomized to one of two chemotherapy regimens. Of 404 patients with RS <12, 348 (86%) received hormonal therapy only. Of the 348 women who received endocrine therapy alone, 110 had HR+, HER2-negative, pN1 breast cancer (Table 2). The endpoints of the trial included the prospective evaluation of the prognostic impact of RS results at a follow-up target of 5 years. The clinical outcomes included DFS and overall survival (OS) in patients with RS <12 treated with endocrine therapy alone. The 5-year DFS in those patients treated with endocrine therapy alone was 94.2% for patients with pN0 and 94.4% in patients with pN1 breast cancer. In a multivariable analysis, fractionally ranked RS result was a significant independent predictor of poorer DFS (*p* = 0.001), along with ≥4N+ breast cancer, tumor grade 3, and tumor size >2 cm. A more recent analysis of the PlanB study showed that 5-year distant disease-free survival (DDFS) was 97.9% for patients with 1–3N+ breast cancer and RS <12 who had endocrine therapy alone.^24^ Nodal status, grade, and RS results were independently prognostic of DDFS in multivariable analysis. Results of the PlanB study confirm the prognostic utility of the RS test among patients with HR+, HER2-negative, N+ breast cancer, although this study used non-standard RS cut-offs of 12 and 25.^23,24,26,31^ (These non-standard cut-offs are similar those used in other prospective clinical studies of the 21-gene test, including TAILORx and RxPONDER; see below.)^32,33^

### Validation of predictive utility of the 21-gene test in HR+, HER2-negative, N+ breast cancer

Prediction of treatment benefit is demonstrated if an interaction exists between the magnitude of treatment effect and the value/level of a biological variable or gene (or a combination thereof). Testing for such an interaction requires that at least two groups (e.g., two arms of a randomized trial) be available for comparison.^34^ This type of analysis was done in a prospectively planned retrospective translational study of tumor samples from the SWOG S8814 trial to test whether a difference in outcome from randomized treatment (tamoxifen alone vs. chemotherapy followed by tamoxifen) depended on the RS result.^18^ The benefit of chemotherapy followed by tamoxifen was found to depend on the RS result: patients with RS ≥31 had significantly better DFS with chemotherapy followed by tamoxifen than with tamoxifen alone (hazard ratio = 0.59; stratified log rank *p* = 0.03; Table 2). DFS was not significantly different for patients with RS <18 who did or did not have chemotherapy. Interaction of chemotherapy benefit with linear RS result was tested, adjusting for number of positive nodes, and was significant over the first 5 years of follow-up (interaction *p* = 0.03). The benefit of chemotherapy (cyclophosphamide, doxorubicin, and fluorouracil (CAF)) was maintained over the entire follow-up period. The test for interaction remained significant after adjustment for age, ethnic origin, tumor size, progesterone receptor status, grade, and TP53 and HER2 levels by immunohistochemistry. A similar significant interaction of treatment with RS results was also found for both the breast cancer-specific survival (BCSS) and OS endpoints.^18^ For BCSS, there was no benefit to CAF in the low or intermediate RS groups; however, for high RS results, 10-year point estimates were 73% for CAF followed by tamoxifen vs. 54% for tamoxifen alone (stratified log-rank *p* = 0.03). Of note, similar to the findings in NSABP B-20 in node-negative disease,^19^ patients with the lowest RS results had numerically worse outcomes with CAF followed by tamoxifen than with tamoxifen alone.

The SWOG S8814 analysis showed that the RS result identifies a group significantly more likely to benefit from chemotherapy. These data represent the strongest evidence available thus far that a genomic assay, namely, the 21-gene test, predicts chemotherapy benefit in HR+, HER2-negative, N+ patients, because the study was prospectively planned to examine this association and was applied to a randomized phase 3 trial with an endocrine therapy-alone arm. The finding of no benefit to chemotherapy in the low RS group was true regardless of the number of positive nodes. In other words, although increasing numbers of positive nodes indeed portend a worse prognosis, the biology predicted by low RS results mandates that endocrine therapy plus non-chemotherapy-based approaches should be pursued in this group of patients to optimize survival.

### Population-based registry reports of outcomes among patients with HR+, HER2-negative, N+ breast cancer treated in routine practice according to RS results

The Surveillance, Epidemiology, and End Results (SEER) program of the National Cancer Institute is an authoritative source of cancer incidence and survival statistics that covers about 30% of the United States population.^35^ In an initial SEER analysis that included 4691 patients with HR+, HER2-negative, N+ breast cancer (defined as micrometastases and 1–3N+) who had RS results, the 5-year estimates of BCSM were significantly different by RS group (*p* < 0.001): 1.0% with RS <18, 2.3% with RS 18–30, and 14.3% with RS ≥31 (Table 3). Adjuvant chemotherapy was reportedly used by 23, 47, and 75% of patients with RS <18, RS 18–30, and RS ≥31, respectively.^27^ A later analysis that included an additional year of follow-up in the SEER registries (*N* = 6768 patients with micrometastastic, 1–3N+, or ≥4N+ breast cancer) confirmed initial findings in the N+ SEER patient population. In particular, 5-year BCSS was 99.1% for patients with RS <18 and micrometastases or 1N+, for whom reported chemotherapy use ranged from 18 to 23%.^28^

Findings from a registry of the Clalit Health Services (CHS), the largest health maintenance organization in Israel, corroborate those of the SEER registry in the US. A CHS registry study examined the relationship between RS results, adjuvant treatment, and outcomes among 709 patients with HR+, HER2-negative, N+ breast cancer (micrometastases and 1–3N+). Among the subset with RS <18 who received no adjuvant chemotherapy (*N* = 342), 5-year distant recurrence was 2.7% and 5-year BCSM was 0.6%. Among all patients with RS <18 (*N* = 379), of whom 7% had adjuvant chemotherapy, 5-year distant recurrence was 1.2% for those with micrometastases and 4.4% with 1N+. In a multivariable analysis that included age, tumor size, tumor grade, nodal status, and RS group, only tumor size (*p* = 0.02) and RS group (*p* = 0.003) were significantly associated with distant recurrence risk.^29^

**Table 3:** Summary of results from prospective, outcomes-based studies of real-world experience

	Standard RS cut-offs		
	RS <18	RS 18–30	RS ≥31
SEER (all N+; *N* = 6768)			
*N* (%)	3919 (64%)	2380 (35%)	469 (1%)
% CT “yes”/“no/unknown”	24%/76%	49%/51%	77%/23%
5-year BCSS (SE)	98.8% (0.3%)	97.3% (0.6%)	88.5% (2.4%)
*p*-value	<0.001		
Clalit (N1mi/1–3N+; *N* = 709)			
*N* (%)	379 (53%)	258 (36%)	72 (10%)
% CT/% no CT	7%/93%	40%/60%	86%/14%
5-year DR (95% CI)	3.2% (1.8%, 5.6%)	6.3% (3.9%, 10.1%)	16.9% (10.0%, 27.9%)
*p*-value	<0.001		
5-year BCSM (95% CI)	0.5% (0.1%, 2.1%)	3.4% (1.7%, 6.7%)	5.7% (2.2%, 14.4%)
*p*-value	<0.001		

*BCSM* breast cancer-specific mortality, *BCSS* breast cancer-specific survival, *CI* confidence interval, *CT* chemotherapy, *DR* distant recurrence, *RS* Recurrence Score result, *SE* standard error

Together, the SEER and CHS registries represent the largest record of outcomes of patients with HR+, HER2-negative, N+ breast cancer who were treated in routine practice based on RS results. Although neither analysis used a randomized design, both were national-level registries that did not exclude patients based on age, sex, comorbidities, or socioeconomic status. The findings of each registry consistently show that patients with RS <18 and N+ breast cancer, particularly micrometastases and 1N+, treated largely without adjuvant chemotherapy had favorable 5-year outcomes. Although the 5-year outcomes of the SEER and CHS registries, as well as the PlanB trial, are relatively short, the meta-analysis by the EBCTCG has shown that the benefit of chemotherapy is seen within the first 5 years after treatment. Importantly, outcomes at 5 years are maintained at 10-year follow-up.^7^

## Discussion

Most clinical practice guidelines recommend that patients with HR+, HER2-negative, N+ breast cancer be considered for adjuvant chemotherapy, because positive nodal status is associated with increased risk of recurrence compared with negative nodal status. Theoretically, if the risk of metastatic recurrence is high enough, very small relative benefits from chemotherapy could translate into meaningful clinical benefits. Evidence suggests, however, that not all patients with N+ breast cancer have poor prognosis nor do all respond equally to chemotherapy. Thus, chemotherapy for all would represent considerable overtreatment for some.

The 21-gene Oncotype DX Breast Recurrence Score test is clinically validated as a prognostic tool that also uniquely predicts chemotherapy benefit in patients with node-negative or N+, HR+, HER2-negative breast cancer.^16–19,26,33^ We have summarized here the body of evidence supporting use of the 21-gene test in the HR+, HER2-negative, N+ setting. RS results provide prognostic insight that augments the information from clinico-pathologic features, including extent of nodal involvement, and further refine risk estimates of distant recurrence. Even in patients with HR+, HER2-negative, N+ breast cancer, who as a cohort were considered at high risk of distant recurrence, the 21-gene test further stratified risk of recurrence based on tumor biology. In multivariable analyses that include clinico-pathologic features, the RS result remained an independent, significant predictor of risk.^23^ Importantly, RS results are calculated based on patient’s tumor biology, thereby sharpening risk estimation to the level of the individual patient.

The idea that prediction of treatment benefit can be inferred from prognosis is flawed. A prognostic factor is one that is associated with clinical outcomes, irrespective of treatment (i.e., either without treatment or with standard treatment).^36^ By contrast, a predictive factor is one that is associated with clinical outcomes *in a treatment-dependent manner*.^36^ A formal statistical test for an interaction between a biomarker and treatment is necessary to determine the predictive utility of that biomarker.^34^ RS results have allowed prediction of chemotherapy benefit in individual patients. The SWOG S8814 analysis showed that patients with RS <18 did not benefit from chemotherapy, in an analysis that was prespecified and therefore designed to address the question of chemotherapy benefit.^18^ Similarly, an NSABP B-20 analysis demonstrated that RS results can predict chemotherapy benefit in patients with node-negative, ER+ breast cancer.^19^ As such, the 21-gene test is the only genomic assay with level 1 evidence of chemotherapy benefit prediction in HR+, HER2-negative breast cancer in both the N+ and node-negative settings.^18,19^

An ongoing study, Treatment for Positive Node, Endocrine Responsive Breast Cancer (RxPONDER; SWOG S1007),^32,37^ should provide further insight into the RS cut-off at which a chemotherapy benefit can be detected in patients with N+ breast cancer. RxPONDER is a phase 3 study in which patients with 1–3N+ breast cancer and RS ≤25 are randomized to hormonal therapy with or without chemotherapy. The primary objective of the RxPONDER study is to measure the interaction between the RS result (as a continuous variable) and chemotherapy. Secondary objectives are to measure time to events (distant recurrence, local/regional recurrence, new invasive primary cancer, or death from any cause). Results of the TAILORx study suggest little-to-no benefit of adjuvant chemotherapy for patients with HR+, HER2-negative, node-negative breast cancer and RS ≤25: excellent outcomes were documented for those with RS <11 assigned to endocrine therapy alone and comparable outcomes for those with RS 11–25 randomized to endocrine therapy ± chemotherapy.^20,33^ In an exploratory analysis, however, some chemotherapy benefit was shown in women ≤50 years with RS 16–25, with the greatest impact on distant recurrence in those with RS 21–25. As a result, RS 25 is now considered the key RS cut-off for node-negative women >50 years and RS 15 for those ≤50 years.

Whether the same holds true in the HR+, HER2-negative, N+ setting remains to be seen. The RS test reveals underlying tumor biology, and as RS-defined risk increases, the degree of chemotherapy benefit also increases. Until results of the RxPONDER study report, the clinical validation studies—SWOG S8814, transATAC, and the prospectively designed PlanB—are supported by the real-world experience of the SEER and CHS registries. Although neither registry was a randomized study, they together showed that thousands of patients with micrometastatic and 1–3N+ breast cancer and RS <18 treated largely or entirely without adjuvant chemotherapy had remarkably favorable outcomes.

In conclusion, the evidence we have summarized is consistent across retrospective-prospective, prospective, and registry studies and shows that patients with N+ breast cancer and a low number of positive nodes who received hormonal therapy but no adjuvant chemotherapy have highly favorable outcomes if they also have low RS results. The data are also consistent across geography, time period of study enrollment, and routine clinical practice, suggesting that the utility of the RS result and its association with outcomes is independent of the treatment practices, time period, or geographic location. This evidence suggests that patients with limited nodal involvement (1–3N+) and low RS results should discuss with their physicians the pros and cons of adjuvant chemotherapy at the time their treatment plans are being decided. Over the last decade or so, clinical guidelines groups worldwide have recognized the value that tumor-specific genomic data bring to treatment decision-making in node-negative early breast cancer. With the steady accumulation of evidence, a role for genomic assays in guidelines for all HR+, HER2-negative early breast cancer, regardless of nodal status, should come into focus.

## Data Availability

Source data for Fig. 1 and Tables 1–3 are provided in the paper. No new datasets were generated or analyzed for this report.
